# Gelatinous Marrow Transformation: A Series of 11 Cases from a Tertiary Care Centre in South India

**DOI:** 10.4274/Tjh.2012.0151

**Published:** 2014-06-10

**Authors:** Sreeya Das, Pritinanda Mishra, Rakhee Kar, Debdatta Basu

**Affiliations:** 1 Jawaharlal Institute of Postgraduate Medical Education and Research (JIPMER), Department of Pathology, Puducherry, India

**Keywords:** Gelatinous transformation, Bone marrow

## Abstract

Gelatinous marrow transformation (GMT) or serous atrophy of bone marrow (BM) is a rare disease characterised by focal marrow hypoplasia, fat atrophy, and accumulation of extracellular mucopolysaccharides abundant in hyaluronic acid. This study reviews 11 cases of GMT from South India. Clinical and haematological parameters, BM aspirate, and biopsies of all patients diagnosed with GMT over a period of 7 years were studied. GMT was diagnosed in BM biopsy based on characteristic morphological appearance and was confirmed by alcian blue positive staining pattern at pH levels of 2.5 and 0.5. Eleven patients were diagnosed with GMT. All were males within the age range of 15 to 50 years. The underlying clinical diagnosis was human immunodeficiency virus positivity in 5 cases, 2 with coexistent disseminated tuberculosis, 1 with cryptococcal meningitis, and 1 with oral candidiasis; disseminated tuberculosis in 1 case; pyrexia of unknown origin in 2 cases; Hodgkin’s lymphoma in 1 case; acute lymphoblastic lymphoma with maintenance chemotherapy in 1 case; and alcoholic pancreatitis in 1 case. BM aspirates showed gelatinous metachromatic seromucinous material in 3 cases. BM biopsies were hypocellular in 7 and normocellular in 4 cases and showed focal GMT in 5 and diffuse GMT in 6 cases. Reactive changes were seen in 4 cases and haemophagocytosis in addition to GMT in 1 case. GMT is a relatively uncommon condition and an indicator of severe illness. It should be differentiated from myelonecrosis, amyloidosis, and marrow oedema. A high index of suspicion is required to diagnose this condition.

## Abstract (tr)

Jelatinöz kemik iliği transformasyonu (JKİT) veya kemik iliğinin (Kİ) seröz atrofisi fokal ilik hipoplazisi, yağ atrofisi ve hyalürinik asitten zengin ekstrasellüler mukopolisakkarit birikimi ile karakterize nadir bir hastalıktır. Bu çalışma Güney Hindistan’dan 11 JKİT olgusunu gözden geçirmektedir. Yedi yıllık periyot boyunca JKİT tanısı alan tüm hastaların klinik ve hematolojik parametreler, Kİ aspirasyon ve biyopsileri incelendi. JKİT tanısı Kİ biyopsisinde karakteristik morfolojik görünüm temel alınarak konuldu, 2,5 ve 0,5 pH düzeyinde pozitif alcian mavi ile boyanarak doğrulandı. On iki hasta JKİT tanısı aldı. Tümü 15-50 yaş aralığında olan erkeklerdi. Altta yatan klinik tanı; 5 olguda insan bağışık yetmezlik virüs pozitifliği olup iki olguda eşlik eden dissemine tüberküloz, bir olguda kriptokokal menenjit ve bir olguda oral kandidiyazis saptandı. Bir olgu dissemine tüberküloz; iki olgu sebebi bilinmeyen yüksek ateş; bir olgu Hodgkin lenfoma; bir olgu idame tedavisi alan akut lenfoblastik lenfoma; ve bir olguda alkolik pankreatit idi. Kİ aspiratları 3 olguda jelatinöz metakromatik seromüsinöz yapı gösterdi. Kİ biyopsileri 7 olguda hiposellüler ve 4 olguda normosellüler idi. Beş olguda fokal JKİT, 6 olguda diffüz JKİT saptandı. Dört olguda reaktif değişiklikler ve bir olguda JKİT’ye ek olarak hemofagositoz izlendi. JKİT nispeten nadir bir durumdur ve ciddi bir hastalığın habercisidir. Miyelonekroz, amiloidoz, ve ilik ödem ile ayırıcı tanısı yapılmalıdır. Tanı için söz konusu durumdan şüphelenilmiş olması gerekir.

## INTRODUCTION

Gelatinous marrow transformation (GMT) or serous atrophy of bone marrow (BM) is a rare disease characterised by focal marrow hypoplasia, fat atrophy, and accumulation of extracellular gelatinous substances [1]. In the 1970s, the gelatinous substances were identified as hyaluronic acid mucopolysaccharides [2]. GMT is not a specific disease of the BM but, like a symptom, it is a morphologic sign of generalised severe illness of a patient. It has been reported to occur in a variety of chronic disorders including prolonged starvation, anorexia nervosa, malabsorption, alcoholism, malignant disease, and human immunodeficiency virus (HIV) infection [1,3,4]. The deposition of gelatinous material and the subsequent alteration in the marrow microenvironment is detrimental to haematopoiesis, which may lead to peripheral haematological abnormalities [5]. We report 11 such cases of GMT. 

## MATERIALS AND METHODS

Eleven cases of GMT, diagnosed at a single tertiary care centre in South India over a 7-year period (January 2005 to September 2011), were retrieved from departmental archives. Clinical information including age, sex, presenting symptoms, physical findings, underlying diseases, treatment history, and HIV status were recorded. Haematological parameters, BM aspiration (BMA), and BM biopsy (BMB) slides were studied. GMT was diagnosed from BMB based on characteristic morphological appearance and was confirmed by alcian blue positive staining pattern at pH levels of 2.5 and 0.5. Informed consent was obtained. 

## RESULTS

**Clinical Details**

All 11 patients were males with ages ranging from 15 to 50 years and a mean age of 30.6 years. The presenting complaints were fever in 4 cases, fever with lymphadenopathy in 4 cases, fever with bleeding manifestation in 2 cases, and abdominal distension in 1 case. History of significant weight loss was present in 6 cases. The clinical and haematological profiles of the cases are given in Table 1. 

The underlying clinical diagnosis was HIV positivity in 5 cases, 2 with coexistent disseminated tuberculosis (TB), 1 with cryptococcal meningitis, and 1 with oral candidiasis; disseminated TB in 1 case; pyrexia of unknown origin (PUO) in 2 cases; Hodgkin’s lymphoma (HD) in 1 case; acute lymphoblastic lymphoma (ALL) with maintenance chemotherapy in 1 case; and alcoholic pancreatitis in 1 case.

Lymphadenopathy was present in 4 cases and mild splenomegaly was present in 1 case. Fine-needle aspiration cytology of lymph nodes was suggestive of lymphoma in 1 case and tuberculous lymphadenitis in 3 cases, of which 2 cases (HIV-positive) showed many acid-fast bacilli (AFB). 

One patient (HIV-negative) was on antituberculous therapy (ATT) and only 1 HIV-positive patient was on antiretroviral therapy (ART) prior to BM examination. The rest of the patients were diagnosed during the course of hospitalisation. The ALL patient was on chemotherapy. One patient who was alcoholic had repeated episodes of pancreatitis and had history of some unknown drug intake. The other patients had no history of any drug intake prior to BM examination. 

In-patient details were available in the latter cases (patients 7-9). A patient with bleeding manifestation (patient 7) received supportive therapy; his symptoms were controlled and he was discharged on request. Patient 9 started ATT during hospitalisation, tolerated it well, and was discharged in stable condition. Patient 10 was started on ART, received antibiotics and antifungal treatment for a palatal ulcer, and was discharged. Patient 11 was scheduled for surgery, which was then deferred due to low counts. He was on conservative therapy and discharged on request. Patient 8, suspected of lymphoma as diagnosed upon fine-needle aspiration cytology, expired during hospitalisation; we subsequently received his postmortem (PM) lymph node and BM biopsies. However, most of the patients were lost to subsequent follow-up.

**Haematological Parameters**

Anaemia was present in all cases with haemoglobin values ranging from 4 g/dL to 10.8 g/dL (mean: 6.9 mg/dL). Five patients in addition had leucopenia. Thrombocytopenia was seen in 6 patients, of whom 3 had a platelet count of less than 40.000/dL. Peripheral smear showed pancytopenia in 2 cases, pancytopenia with iron deficiency anaemia in 1 case, bicytopenia in 2 cases, and eosinophilia in 1 case.

**Figure 1:**
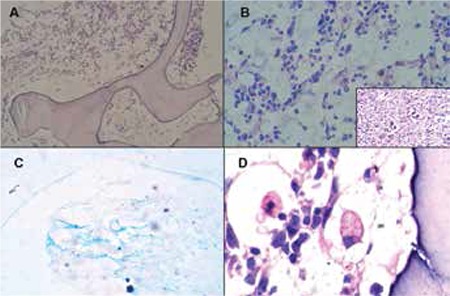
Bone marrow biopsy (patient 8) showing focal GMT (H&E, 100x). B) Higher magnification of the same showing gelatinous material, which is pale blue in colour (H&E, 400x); inset: lymph node biopsy of the same patient showing Reed-Sternberg cells and mummified cells (H&E, 400x). C) The seromucinous material is positive with alcian blue (400x). D) Bone marrow biopsy also showing haemophagocytosis (H&E, 1000x).

BMA and BMB were done in 10 cases, and in 1 case only PM biopsy was done. Clinical indications for BM were PUO with cytopenia (2 cases), suspected lymphoma (2 cases), bicytopenia (2 cases), pancytopenia (2 cases), neutropenia (1 case), disseminated TB (1 case), and PM BM biopsy (1 case). The PM case was suspected as lymphoma clinically and was confirmed to be classical HD (lymphocyte-depleted variant) upon PM lymph node biopsy (Figure 1b, inset). 

**Figure 2:**
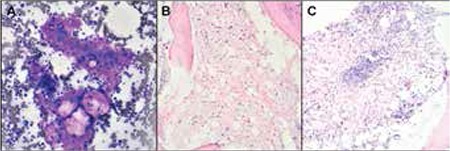
Bone marrow aspirate showing metachromatic seromucinous material with a few entrapped fat spaces (Giemsa, 100x). B) Bone marrow biopsy showing diffuse GMT (H&E, 100x). C) Bone marrow biopsy from HIV-positive patient (patient 10) showing a reactive lymphoid nodule with focal GMT (H&E, 100x).

BMA was unsatisfactory in 7 cases and in 3 cases there was reactive marrow. In 3 cases, gelatinous metachromatic seromucinous material was seen focally, with a few entrapped haematopoietic cells (Figure 2a).

BMBs were hypocellular in 7 cases and normocellular in 4 cases; they showed focal GMT in 5 cases (Figures 1a and 1b) and diffuse GMT in 6 cases (Figure 2b). Under low-power examination, GMT appeared as amorphous, finely granular, light blue to pale pink material, which was stained by alcian blue at pH 2.5 (Figure 1c). A variable degree of atrophy of fat cells was seen. Reactive changes in the form of plasmacytosis, histiocytic prominence, lymphoid aggregate (Figure 2c), and secondary dysplasia were seen in 4 cases. The PM case showed evidence of haemophagocytosis in addition to GMT (Figure 1d).

## DISCUSSION

GMT has also been termed as ‘starvation marrow’ or serous (fat) atrophy. Chronic malnutrition may be the source of GMT because it is very commonly seen in anorexia, starvation, and other malnourished states [6]. Wang et al. [7] described a case of GMT in a patient with a starch-free diet, which was reversible after restoration of a normal diet. Hyaluronic acid, a ubiquitous component of the extracellular matrix, plays an important role in repairing damaged tissue. It is suggested that hyaluronic acid may be a substance to replace fat cells in the marrow that are used in catabolic states of disease. However, since most patients with chronic wasting disease do not show GMT, additional factors besides fat cell mobilisation may be necessary for the development of GMT [1].

GMT has a male predominance and is usually rare in children. Only rare case reports are available showing GMT in children [8]. In our study, all patients were males and the youngest was 15 years of age. In a large series conducted by Bohm [1] on 158 patients, GMT was found in all age groups except children. On the other hand, in a study from India, Jain et al. [9] observed GMT in 14 children (total cases: 43), the youngest being a child of 6 months old with cholestatic jaundice. 

The spectrum of underlying disease in GMT is heterogeneous. The most common clinical association in our series was HIV positivity (5 cases), followed by PUO (2 cases), which is similar to the findings in previous studies [1,5]. Four previously published cases of GMT in patients with HIV positivity [10] were also included in this study. However, in a larger case series of 43 cases from North India [9], none of the cases were associated with HIV infection. In another study by Sen et al. [3] of 65 cases, GMT was most commonly associated with infections. Bohm [1] found association with 1 disease in most cases, but in 27 (17%) cases, 2 or 3 diseases capable of inducing GMT were present. Similarly, in our study, in 2 cases the underlying pathology was HIV with disseminated TB. 

GMT has also been described in association with malignancies like leukaemia, lymphoma, metastatic deposits, and multiple myeloma, with or without chemotherapy [1,3,6]. Recently it was described with imatinib therapy [11]. In our study, 1 ALL patient was on chemotherapy and 1 HD patient was not on therapy. It has been suggested that some malignant cells might produce or stimulate the production of hyaluronic acid, leading to GMT [12]. 

GMT is almost always associated with anaemia. In the study by Jain et al. [9], all patients had moderate to severe anaemia. In Bohm’s study [1], 82% of patients were anaemic and 78% had severe weight loss or cachexia. However, the degree of anaemia did not correlate with the extent of GMT in the marrow. Similarly, all our patients had moderate to severe anaemia, but the degree of anaemia did not correlate with the extent of GMT. Five patients had leucopenia and 6 patients had thrombocytopenia, which did not show any correlation with the degree of GMT. This is similar to the findings of previous studies [1]. 

Most of the GMT lesions could be diagnosed from BMB sections with haematoxylin and eosin (H&E) staining. BM aspirate was diluted in 7 cases and 3 cases showed cellular reactive marrow; however, those 3 cases showed focal GMT in the BMB. Gelatinous seromucinous material was seen only in 3 cases in BMA. In a large series of cases, Bohm [1] diagnosed GMT in all cases (158) from BMB sections. In another series of cases, however, 24 out of 43 cases were diagnosed with BMA, 17 cases with both BMA and BMB, and 2 cases with only with BMB [9].

Under low-power examination, GMT appears as a hypocellular area with mild to marked hypoplasia of ematopoietic cells. There is atrophy of fat cells, which are both reduced in number and of variable size. Both fat and haematopoietic cells are replaced by amorphous material, which has a light blue to pale pink and finely granular appearance. This is stained by alcian blue at pH 2.5. The gelatinous material must be differentiated from marrow necrosis, oedema, and amyloidosis [9]. Necrosis is granular and may be associated with necrosis of the adjacent bone. Oedema is differentiated by absence of fat cell atrophy. Amyloidosis is homogeneous and can be excluded by Congo red staining. However, none of the conditions stain positively with alcian blue. 

One patient was diagnosed with HD in a PM biopsy, and the BM showed evidence of haemophagocytosis along with focal GMT. GMT can be associated with HD [9]. Similarly, haemophagocytosis is also known to occur in association with HD [13]. All these changes are described individually in association with HIV [14]. However, in this patient, HIV status was negative. Coexistence of both GMT and haemophagocytosis in an HIV-negative patient with HD with a fatal course has not been reported before, to the best of our knowledge.

## CONCLUSION

GMT is a relatively uncommon condition and an indicator of severe illness. It should be differentiated from myelonecrosis, amyloidosis, and marrow oedema. A high index of suspicion is required to diagnose this condition. 

## CONFLICT OF INTEREST STATEMENT

The authors of this paper have no conflicts of interest, including specific financial interests, relationships, and/or affiliations relevant to the subject matter or materials included. 

## Figures and Tables

**Table 1:**
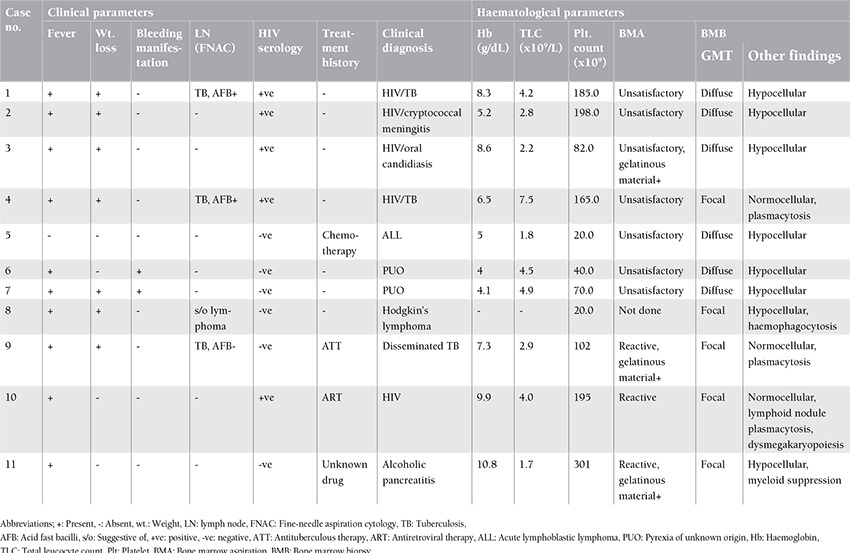
Clinico-haematological profile of all patients.
